# Resveratrol-Based Nanoformulations as an Emerging Therapeutic Strategy for Cancer

**DOI:** 10.3389/fmolb.2021.649395

**Published:** 2021-09-01

**Authors:** Javad Sharifi-Rad, Cristina Quispe, Zhazira Mukazhanova, Ewa Knut, Aknur Turgumbayeva, Aliya Kipchakbayeva, Gulnaz Seitimova, Mohamad Fawzi Mahomoodally, Devina Lobine, Aaron Koay, Jinfan Wang, Helen Sheridan, Gerardo Leyva-Gómez, María L. Del Prado-Audelo, Hernán Cortes, Antonio Rescigno, Paolo Zucca, Oksana Sytar, Muhammad Imran, Célia F. Rodrigues, Natália Cruz-Martins, Halina Ekiert, Manoj Kumar, Ahmad Faizal Abdull Razis, Usman Sunusi, Ramla Muhammad Kamal, Agnieszka Szopa

**Affiliations:** ^1^Phytochemistry Research Center, Shahid Beheshti University of Medical Sciences, Tehran, Iran; ^2^Facultad de Ciencias de la Salud, Universidad Arturo Prat, Iquique, Chile; ^3^Department of Natural Sciences and Technologies, Sarsen Amanzholov East Kazakhstan State University, Ust-Kamenogorsk, Kazakhstan; ^4^Chair and Department of Pharmaceutical Botany, Faculty of Pharmacy, Jagiellonian University, Medical College, Kraków, Poland; ^5^Asfendiyarov Kazakh National Medical University, School Pharmacy, Almaty, Kazakhstan; ^6^Al-Farabi Kazakh National University, Higher School of Medicine, Almaty, Kazakhstan; ^7^Faculty of Chemistry and Chemical Technology, Al-Farabi Kazakh National University, Almaty, Kazakhstan; ^8^Department of Health Sciences, Faculty of Medicine and Health Sciences, University of Mauritius, Réduit, Mauritius; ^9^Trinity College Dublin, NatPro (Natural Products Research Centre), School of Pharmacy and Pharmaceutical Science, Dublin, Ireland; ^10^Departamento de Farmacia, Facultad de Química, Universidad Nacional Autónoma de México, Ciudad de México, Mexico City, Mexico; ^11^Laboratorio de Medicina Genómica, Departamento de Genética, Instituto Nacional de Rehabilitación Luis Guillermo Ibarra Ibarra, Ciudad de México, Mexico City, Mexico; ^12^Department of Biomedical Sciences, University of Cagliari, Cittadella Universitaria, Cagliari, Italy; ^13^Department of Plant Biology, Institute of Biology, Taras Shevchenko National University of Kyiv, Kyiv, Ukraine; ^14^Department of Plant Physiology, Slovak University of Agriculture, Nitra, Slovakia; ^15^University Institute of Diet and Nutritional Sciences, The University of Lahore, Lahore, Pakistan; ^16^Laboratory for Process Engineering, Environment, Biotechnology and Energy—Department of Chemical Engineering, Faculty of Engineering, University of Porto, Porto, Portugal; ^17^Faculty of Medicine, University of Porto, Porto, Portugal; ^18^Institute for Research and Innovation in Health (i3S), University of Porto, Porto, Portugal; ^19^Laboratory of Neuropsychophysiology, Faculty of Psychology and Education Sciences, University of Porto, Porto, Portugal; ^20^Chemical and Biochemical Processing Division, ICAR – Central Institute for Research on Cotton Technology, Mumbai, India; ^21^Department of Food Science, Faculty of Food Science and Technology, Universiti Putra Malaysia, Selangor, Malaysia; ^22^Natural Medicines and Products Research Laboratory, Institute of Bioscience, Universiti Putra Malaysia, Selangor, Malaysia; ^23^Department of Biochemistry, Bayero University Kano, Kano, Nigeria; ^24^Department of Pharmacology, Federal University Dutse, Dutse, Nigeria

**Keywords:** resveratrol, cancer therapy, drug-delivery systems, clinical applications, bio-availability

## Abstract

Resveratrol is a polyphenolic stilbene derivative widely present in grapes and red wine. Broadly known for its antioxidant effects, numerous studies have also indicated that it exerts anti-inflammatory and antiaging abilities and a great potential in cancer therapy. Regrettably, the oral administration of resveratrol has pharmacokinetic and physicochemical limitations such as hampering its effects so that effective administration methods are demanding to ensure its efficiency. Thus, the present review explores the published data on the application of resveratrol nanoformulations in cancer therapy, with the use of different types of nanodelivery systems. Mechanisms of action with a potential use in cancer therapy, negative effects, and the influence of resveratrol nanoformulations in different types of cancer are also highlighted. Finally, the toxicological features of nanoresveratrol are also discussed.

## Introduction

Molecular targeted therapy has been developed as a promising tool to overcome the lack of specificity of chemotherapeutic agents, and in such a framework, nanotechnology emerges as a powerful strategy (Gu et al., 2009; Sanna et al., 2013a). Presently, several smart drug delivery systems, such as carbon-based and polymeric materials, metallic nanoparticles, or liposomes, have been adopted for cancer treatment. In addition, a growing emphasis has been placed on naturally occurring bioactive compounds for chemotherapy and chemoprevention in several kinds of cancer, and secondly, there has been a more pronounced focus on nanotherapy where the proof-of-concept of cancer prevention using nanoformulations is in a stage of rapid development (Navya et al., 2019). Different nanoformulations have been compared to the already available anticancer drugs; among other aspects, they have revealed to be more soluble, stable, effective, and improved biodistribution pattern. However, careful attention must be paid in the development of effective targeted formulations that can contribute to raise therapeutic outcomes while exerting no significant damage to the tissues (Revia and Zhang, 2016; Navya et al., 2019).

Among active natural compounds, polyphenols are gaining high popularity because of their bioavailability traits and promissory beneficial effects, including antioxidant, anti-inflammatory, antiaging, and anticancer properties (Upadhyay and Dixit, 2015; Karakaya et al., 2019). Indeed, the role of polyphenols as oxidative stress modulators in cancer has been deeply discussed (Mileo and Miccadei, (n.d.)). However, considering their low stability as a limiting factor, the topical application of nanopolyphenols is thought to represent a valuable technology to overcome some limitations in pharmacokinetics, targeting efficacy, and safety concerns (Menaa et al., 2014). Among this broad class of compounds, resveratrol has gaining a key scientific interest due its good anticancer and antioxidant effects (Summerlin et al., 2015). Despite its isolation in 1939 firstly from the roots of the Japanese plant *Polygonum cuspidatum* (Timmers et al., 2012), resveratrol is an important antioxidant present abundantly in red grapes, red wine, and a variety of other dietary sources, such as peanuts, raspberries, blueberries, and mulberries (Chong et al., 2009; Jasiński et al., 2013; Weiskirchen and Weiskirchen, 2016; Jeandet et al., 2021).

**Figure 1:**
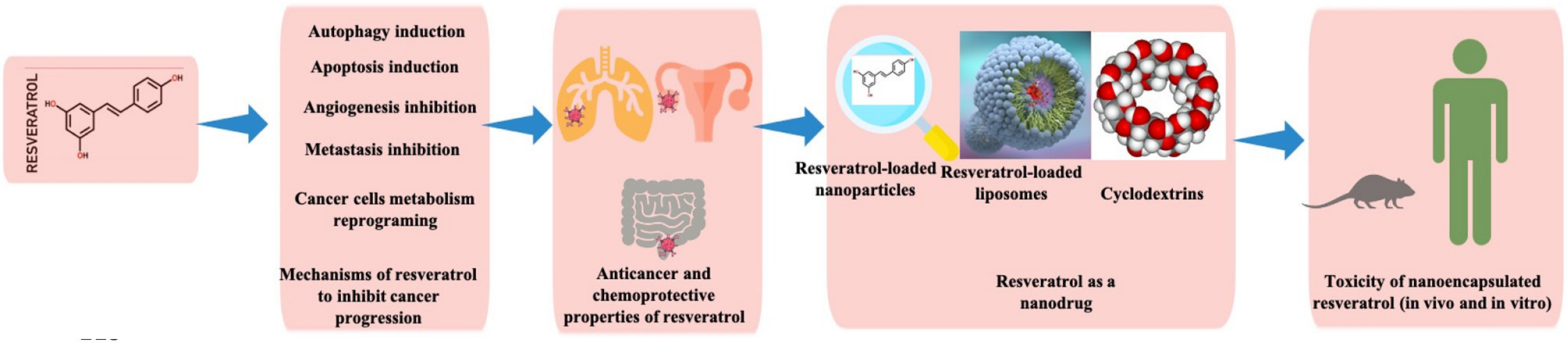
Illustration showing various components discussed in the current review.

This molecule is a natural stilbenoid with the chemical formula 3,5,4′-trihydroxy-trans-stilbene. Interestingly, resveratrol is a plant secondary metabolite, phytoalexin, produced as a result of the adaptive reaction to environmental stress factors, such as fungal infections, injury, and UV irradiation (Langcake and Pryce, 1976; Bhat et al., 2001a; Jeandet et al., 2010; Aluyen et al., 2012; Summerlin et al., 2015). With a broad action as an antioxidant (Öztürk et al., 2017), resveratrol has shown excellent chemopreventive and chemotherapeutic effects against certain types of cancers (Figure 1).

In a tumor microenvironment, resveratrol has shown to directly interfere with cancer initiation, promotion, and progression (significant stages of carcinogenesis) (Aggarwal et al., 2004; Summerlin et al., 2015; Velmurugan et al., 2018). In this respect, resveratrol stimulates the apoptosis of tumor cells, prevents tumor-derived nitric oxide synthase expression, blocks tumor growth and migration, and also inhibits the cyclooxygenase (COX) activity (Aggarwal et al., 2004). These specific mechanisms have been corroborated in different types of cancer, and recent studies have suggested that resveratrol may also be useful when given in combination with other agents (Singh et al., 2013). Indeed, the proposed mechanisms of action through which resveratrol exerts its anticancer effects involve multiple pathways. For example, based on tumor cell lines, the anti-proliferative and pro-apoptotic activities of resveratrol include the ability to modulate the expression of pro-oncogenic and tumor suppressor microRNAs (miRNAs), peroxisome proliferator-activated receptor (PPAR), nuclear factor-kappa B (NF-kB), nuclear respiratory factor- (NRF-) 1 and 2, gamma coactivator 1 alpha (PGC-1α) and p53 transcription factors, and transforming growth factor β (TGF-β) signaling pathways, to exert pro-di?erentiation abilities and act in synergy with conventional anticancer drugs (Vervandier-Fasseur and Latruffe, 2019). However, despite such excellent abilities (de Oliveira et al., 2019; Zhang et al., 2019), resveratrol has several limitations, such as low oxidative stability, poor water solubility, and high photosensitivity, which greatly restrict their application. Hence, nanotechnology can be an interesting technology to prevail these restraints (Ahmadi et al., 2019).

In this sense, the current review discusses the resveratrol anticancer mechanisms and gives a key emphasis on the potential use of its nanoformulations as an emerging cancer therapy, through the application of different kinds of nanodelivery systems.

## Mechanisms of Resveratrol to Inhibit Cancer Progression

### Autophagy Induction

Autophagy is a self-destructive process (Glick et al., 2010) to ensure the survival or boost the death of altered/damaged cells, anticipating the environments of stress, damage, starvation, aging, and pathogen infection. In addition, autophagy also aids to remove mis-folded or aggregated proteins, depurate damaged organelles, and eliminate intracellular pathogens (Elshaer et al., 2018).

A reasonable strategy for the possible application of resveratrol in cancer treatment is through its ability to induce autophagic cell death by the overstimulation of autophagy in apoptosis-defective cells. The most well-known signaling pathways for resveratrol include the induction of autophagy by an increased activation and expression of sirtuin1 (SIRT1), the inhibition of protein kinase B/mammalian target of rapamycin (Akt/mTOR), and the activation of p38-mitogen-activated protein kinase (MAPK) as observed in cases of non-small-cell lung cancer (NSCLC) (Wang et al., 2018). In addition, it has been shown that a SIRT1 inhibitor (nicotinamide: 5 mmol/L) and an autophagy inhibitor (3 methyl adenine: 10 mmol/L) enhance the anti-tumor activity of resveratrol at 200 μM. Specifically, in head and neck cancer, a deficient autophagy triggers p62 accumulation (an autophagy adapter protein) (Tian et al., 2019), with resveratrol being able to stimulate autophagy and accelerate the p62 degradation, in addition to inhibiting the nuclear factor erythroid 2-related factor 2/antioxidant response element (Nrf2/ARE) pathway. In breast carcinoma, resveratrol led to an increase in phosphatase and tensin homolog (PTEN) protein levels while decreasing the Akt phosphorylation. In prostate and lung cancers (LCs), resveratrol incites a downregulation of stromal interaction molecule 1 (STIM1) and an inactivation of the mTOR pathway whereas in colorectal cancer, it boosts autophagy by increasing reactive oxygen species (ROS) production and inducing caspases-8 and−3 (Miki et al., 2012). In oral cancer, it triggers autophagy by increasing AMP-activated protein kinase (AMPK) phosphorylation (Chang et al., 2017), whereas in ovarian cancer, it stimulates Beclin-1 (Opipari et al., 2004). Finally and noteworthy, an excessive autophagic damage triggers cell death (Alayev et al., 2015).

### Apoptosis Induction

In cellular regulative mechanism, resveratrol has revealed to be effective to induce apoptosis by binding with α_v_β_3_ integrin, to activate extracellular signal-regulated kinase (ERK)1/2 *via* MAPK-kinase [mitogen-activated extracellular signal-regulated kinase (MEK1/2)] (Elshaer et al., 2018), an essential protein in the interaction of cancer cells. Yet, this have been reported to occur only at specific concentrations (1 pM−10 μM) and for short-term activation (Lin et al., 2011b). Being confirmed as the main mechanism in breast, prostate, ovarian, glial, head, and neck cancer cells (Lin et al., 2002, 2008a,b, 2011a; Zhang et al., 2004; Yang et al., 2011), other researchers have also shown that in addition to MAPK, resveratrol also intervenes in p38 kinase and cJun N-terminal kinase (Dong, 2003). Interestingly, it was also reported that the activated form of ERK1/2 induces the phosphorylation of p53 in several human cancer cell lines (Lin et al., 2011b), with resveratrol being able to induce p53-independent apoptosis. Indeed, the p53 oncogene suppressor protein is involved in apoptosis, having the ability to be coupled to DNA in its active form. In addition, resveratrol at a dose of 10 μM is able to boost the nuclear accumulation of COX-2 by p53-dependent apoptosis in breast, prostate, ovarian, head, and neck cancer cells (Lin et al., 2008a,b, 2011a). Specifically, the inducible COX-2 associated with small ubiquitin-related modifier-1 (SUMO-1) is transported to the nucleus and forms a complex with phosphorylated p53, and phosphorylated ERK1/2 and p300. These processes are activated in an ERK1/2 and a COX-2-dependent way. Moreover, inducible nuclear-accumulated COX-2 potentiates resveratrol-induced p53-dependent apoptosis.

### Angiogenesis Inhibition

Angiogenesis is a necessary mechanism for cancer growth and development as cancer cells require blood vessels for nutrients and oxygen for its growth and metastasis. Increases in the expression and secretion of matrix metalloproteinases (MMP) play a key role in angiogenesis. In this regard, resveratrol is able to inhibit endothelial cell adhesion and migration by reducing an MMP-2 activity during neo-angiogenesis in both *in vivo* and *ex vivo* assays (Cao et al., 2005). This mechanism is particularly valuable in chemopreventive aspects. Other antiangiogenic activities performed by resveratrol include the inhibition of hypoxia-inducible factor-1alpha (HIF-1alpha) accumulation, an increased expression of thrombospondin-1 (TSP1), a natural inhibitor of angiogenesis (Trapp et al., 2010). Specifically, the treatment of melanoma cell lines (i.e., A375, M14, and YUZAZ6) with resveratrol for 72 h revealed a dose-dependent decrease in metabolic activity as measured by the XTT assay (Figure 1), with A375 cells revealing to be the most sensitive (IC_50_ = 28.79 μM), followed by YUZAZ6 cells (IC_50_ = 36.46 μM), and M14 cells (IC_50_ = 103.44 μM), which were the most resistant ones (Trapp et al., 2010). In addition, after a three-dimensional spheroidal co-culture, the treatment of resveratrol (50 μM, 48 h) led to a decrease in endothelial cell viability when grown in a coculture with A375, YUZAZ6, or WM3211 melanoma cell lines. Resveratrol decreases the expression of prostaglandins (PGs) by the inhibition of a COX-2 enzyme, which catalyzes the arachidonic acid conversion into PGs. In addition, both PGs and NO play an important role in cell proliferation and angiogenesis, which trigger tumor growth and metastasis (Trapp et al., 2010).

### Metastasis Inhibition

Metastasis is the process of spreading cancer cells to healthy organs, usually through the blood or lymph. In this respect, resveratrol has shown to be able to decrease the expression levels of MMP-2 and MMP-9, fibronectin, α-smooth muscle actin (α-SMA), phosphorylated phosphatidylinositol 3-kinase (P-PI3K), phosphorylated-Akt (P-Akt), mothers against decapentaplegic homolog (Smad)2, Smad3, phosphorylated (P)-Smad, P-Smad3, vimentin, Snail1, and Slug while decreasing E-cadherin levels in MDA-MB-231 human breast cancer cells (Sun et al., 2019). These markers are characteristic of the TGF-β1-induced epithelial-mesenchymal transition (EMT), with resveratrol being able to inhibit the MDA231 cell migration *via* EMT. In a study, it was stated that MDA231, MDA-MB-453 (MDA453), MDA-MB-436 (MDA436), and BT549 (BT-549) cells treated with distinct concentrations of resveratrol for 3 days evidenced different patterns of cell survival. In 3-(4,5-dimethylthiazol-2-yl)-5-(3-carboxymethoxyphenyl)-2-(4-sulfophenyl)-2H-tetrazolium (MTS) assay, cells treated with resveratrol at concentrations of 12.5, 100 μM for 48 h and 72 h presented a significant decrease in the survival rate compared to control cells. Using the transwell migration assays, resveratrol at concentrations of 12.5, 25, and 50 μM inhibited the migration of MDA231 cells, with the cell migration inhibition degree being concentration-dependent. Importantly, the EMT pathway has been often associated with tumor invasion and metastasis in ovarian, breast, colon, lung, prostate, oral, liver cancer, etc. (Guarino, 2007).

### Cancer Cell Metabolism Reprograming

**Figure 2:**
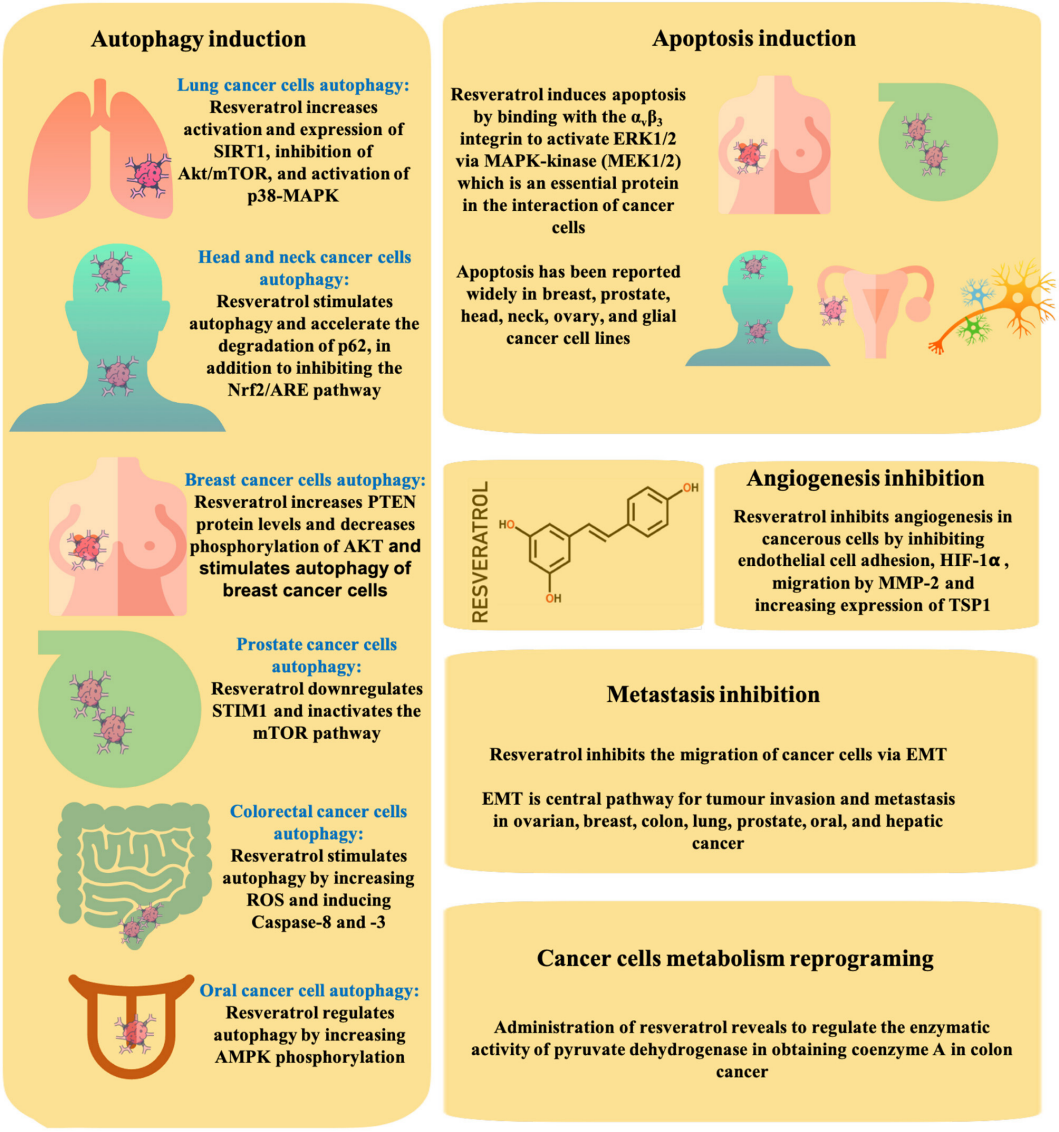
Dominant pathways by which resveratrol inhibits cancer progression, including the induction of autophagy and apoptosis, inhibition of angiogenesis and metastasis, and reprogramming of the cellular metabolism.

In cancer cells, there is an imbalance of metabolism, mainly in energy consumption-related pathways. Particularly, a marked change in glucose metabolism toward ATP generation has been progressively suggested (Elshaer et al., 2018). The administration of resveratrol has been shown to regulate the enzymatic activity of pyruvate dehydrogenase (PDH) in obtaining coenzyme A in colon cancer (Saunier et al., 2017). As main findings, the resveratrol-induced metabolic changes were described as related to the modifications at the level of expression of key proteins involved in glucose metabolism. In addition, it was stated that resveratrol did not modulate the glucose transporter 1 (GLUT1), rate-limiting enzyme of pentose phosphate pathway, i.e., glucose 6-phosphate dehydrogenase (G6PD), the enzyme that catalyzes the last step of the glycolysis pyruvate kinase M2 (PKM2), or lactate dehydrogenase A (LDHA), which converts pyruvate to lactate. The activity of the PDH complex was also measured in colon cancer cells following the treatment with 10 μM resveratrol for 48 h using [^14^C_1_]-pyruvate, and it was observed that the activity of PDH complex was enhanced by 2.6-fold. Various mechanisms involved in the ability of resveratrol to inhibit cancer progression are presented in Figure 2.

## Complementary Confirmation Research of Anticancer Properties

### Inhibition of Lung Cancer

According to the WHO report, LC is the most common type of cancer worldwide. For men, this cancer represents the leading cause of death, whereas for women it is the second one. Noteworthy, there were 1.76 million deaths worldwide due to LC in 2018, and despite the advances stated in medicine and technology, the overall 5-year survival rate is <16% (Mao, 2016; WHO, 2018). Based on these data, the need for novel therapies and treatments for LC is evident.

**Figure 3:**
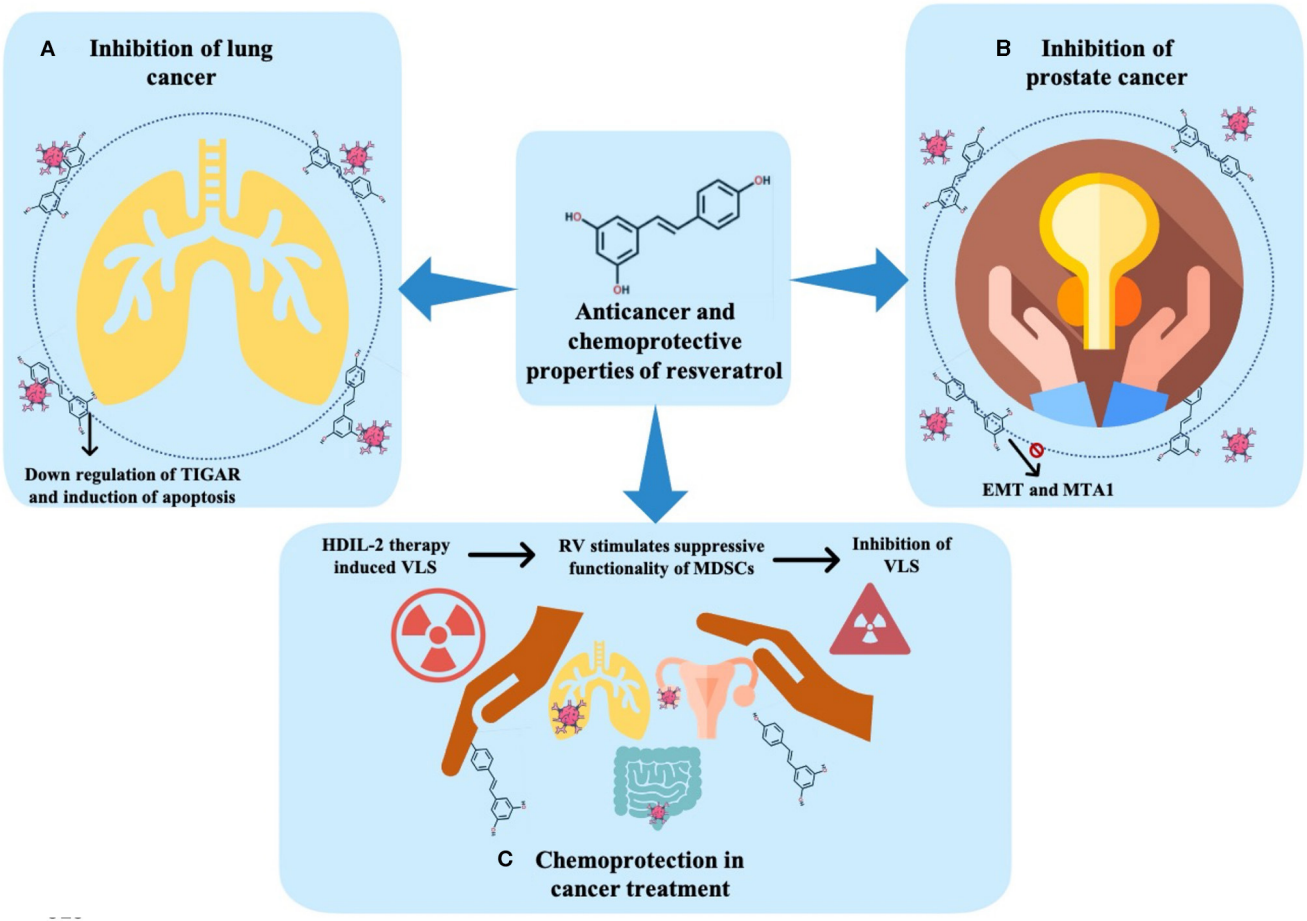
Anticancer and chemoprotective effects of resveratrol. **(A)** Inhibition of lung cancer. **(B)** Inhibition of prostate cancer. **(C)** Chemoprotection in cancer treatment. TIGAR, tumor protein (TP) 53-induced glycolysis and apoptosis regulator; EMT, epithelial-mesenchymal transition; MTA, metastasis-associated protein; HDIL-2, high-dose interleukin-2; RV, resveratrol; MDSCs, myeloid-derived suppressor cells; VLS, vascular leak syndrome.

In this regard, some authors addressed the ability of resveratrol to induce cell senescence as the doses required for senescence are lower than that needed to achieve apoptosis (Luo et al., 2013). As main findings, at a low dose (10–50 μM), in human NSCLC, resveratrol led to an increase in the expression of p53 and p51, and β-galactosidase (associated with senescence). Thus, the mechanisms by which resveratrol could induce premature senescence have been reported to be related to the increment in both DNA double-strand breaks and ROS production. Similarly, in another study, the effect of resveratrol on ROS production in human NSCLC cells (H129 cells) as well as in a breast cancer cell line (MCF-7 cells) was analyzed. It was concluded that, in both the lines, resveratrol treatment significantly increased the ROS levels while reducing the intracellular glutathione (GSH) levels (Kumar et al., 2015). Likewise, the authors evaluated the effect of resveratrol on the tumor protein (TP)53-induced glycolysis and apoptosis regulator (TIGAR). As main findings, 48 h after resveratrol treatment, both cell lines presented a dose-dependent decrease in TIGAR protein through the downregulation of mTOR signaling and an increment in Poly(ADP-ribose) polymerase (PARP) cleavage, considered the hallmark of an increase in cell death and apoptosis (Figure 3A) (Kumar et al., 2015).

### Inhibition of Prostate Cancer

Prostate cancer (PC) is the second most common cancer type in male subjects. Usually, it is treatable at early stages, however, there are over 2,50,000 deaths worldwide among clinically advanced cases. Genetic studies have shown that PC is related to DNA alterations triggering dysregulation in genes involved in PC development. Mutations in forkhead box A (FOXA)1, speckle-type POZ protein (SPOP), and TP53 as well as copy number alterations in MYC, PTEN, and retinoblastoma (RB)1 are the examples of recurrent modifications in PC (Joniau et al., 2014; Abstract and Brief, 2015; Robinson D. et al., 2015).

In this way, resveratrol has been proposed as a good therapeutic strategy in PC. Various studies have addressed the influence of resveratrol in PC through addressing its effects on EMT, a process associated with the progression of the disease (Li et al., 2014). As main findings, the authors observed a decrease in cell proliferation in the presence of resveratrol. In addition, the mesenchymal cell phenotype was less evident compared to control cells (without resveratrol). Thus, it was suggested that the anticancer properties of resveratrol could be linked to the ability to inhibit EMT, probably by the deactivation of a hedgehog signaling pathway (Li et al., 2014). Similarly, the effect of resveratrol in PC was also explored to elucidate the mode of action of this compound, being observed a critical downregulation of metastasis-associated protein (MTA1), which is highly related to the repressive chromatin involved with cancer progression and metastasis in three PC cell lines (Figure 3B) (Kai et al., 2010). Moreover, the modifications in the expression of miRNAs in PC include the overexpression and amplification of oncogenic miR-17~92 and miR-106b~25 clusters. In addition, it has been reported that some of these clusters are acting as a target to the tumor suppressor gene PTEN, which is often affected in PC. More recently, some data have revealed that both resveratrol and its potent natural analog, pterostilbene, are efficient in restoring the tumor suppressor gene PTEN, and might thus be viewed as an attractive miRNA-mediated chemopreventive and therapeutic agent (Dhar et al., 2015).

### Chemoprotection in Cancer Treatment

Chemical therapy, radiation therapy, or immunotherapy are the most common therapeutic approaches used for cancer treatment. Despite the approaches being highly effective, they are also linked to significant undesired side effects, such as fatigue, insomnia, vascular syndromes, and cell injury (Mughal, 2010; Bower and Lamkin, 2013; Safarzadeh et al., 2014).

The search for chemoprotective molecules has become even more relevant in the last few years, with the use of resveratrol for chemoprotective purposes being increasingly reported as useful (Guan et al., 2012; Xu et al., 2012). For example, the interleukin- (IL-) 2 therapy promotes endothelial cells injury and induces vascular leak syndrome (VLS). Guan et al. (2012) analyzed the protective action of resveratrol in endothelial cells before high-dose IL-2 (HDIL-2) treatment. In that study, female C57BL/6 mice with VLS induced by HDIL-2 and treated with B16F10 melanoma cell line for tumor cell implantation were exposed to resveratrol. The results indicated that the treatment that begins 1 day after VLS induction and tumor implantation inhibited the expansion of VLS trigger by HDIL-2 therapy, possibly related to the ability of resveratrol to stimulate the suppressive functionality of myeloid-derived suppressor cells (MDSCs). MDSCs play a strategic role in suppressing the development of VLS. Additionally, cells were treated with resveratrol, IL-2, or both. The authors found that resveratrol inhibited the VLS and stimulated the expression of arginase (Arg) 1. Conversely, the IL-2 treatment did not inhibit the VLS development. Interestingly, the use of resveratrol in combination with HDIL-2 was more effective than the use of HDIL-2 alone, leading to a marked inhibition in LC growth (Figure 3C) (Guan et al., 2012).

## Possible Adverse Effects of Resveratrol in Cancer

As described above, the anticancer properties of resveratrol are categorical, being stated a high potential to be employed as a co-adjuvant therapy for numerous types of cancer. However, some questions concerning the possible harmful effects of this nutraceutical still remain unanswered (Salehi et al., 2018). Indeed, although it has been shown that several phytochemicals, including resveratrol, exhibit a biphasic dose-response (Calabrese et al., 2010; Mukherjee et al., 2010; Jodynis-Liebert and Kujawska, 2020), this response is characterized by an opposite biological effect at different concentrations, a phenomenon called hormesis (Calabrese and Baldwin, 2002). For example, several studies have shown that resveratrol exerts biphasic effects on both the viability and proliferation of diverse cell lines (Tvrdá et al., 2015; Kumar et al., 2016; Plauth et al., 2016; San Hipólito-Luengo et al., 2017). A study demonstrated that low doses of resveratrol augment the viability of HepG2 (1–100 μM), normal human dermal fibroblasts (NHDF) (1–300 μM), and normal human epidermal keratinocyte (NHEK) (<50 μM) cell lines by 15%, 15%, and 20%, respectively (Plauth et al., 2016). In contrary, the doses of 500 μM of resveratrol led to a significant reduction on cell viability, i.e., 40% for HepG2 and 75% for NHDF and NHEC cells. According to the authors, this dual response could be due to the generation of oxidation products and increases in the expression of some cellular defense genes (Plauth et al., 2016). Another study found that, at 1, 10, and 20 μM, resveratrol increased the proliferation of neural progenitor cells by 10%, 35%, and 25%, respectively. Interestingly, at the doses of 50 and 100 μM, it produced an opposite effect, reducing cell proliferation by 50% and 65%, respectively. The authors claimed that these contrary effects are mediated by changes in the phosphorylation of p38 kinases and ERKs (Kumar et al., 2016). Likewise, a recent manuscript revealed similar trends following the use of resveratrol in colon cancer cell proliferation. As main findings, at the concentrations of 1–10 μM, resveratrol enhanced cell proliferation after 96 h of treatment, whereas at 50 and 100 μM it decreased the cell number, suggesting cytotoxicity. This effect was proposed by the authors as being mediated by the activation of nicotinamide adenine dinucleotide phosphate (NADPH) oxidase and to an increase in histone gamma (H2A) histone family member X (H2AX) (San Hipólito-Luengo et al., 2017). Interestingly, at low concentrations (1–50 μM), resveratrol diminished the levels of superoxide products, whereas at higher concentrations (100 and 200 μM) it markedly increased the production of superoxide. On the other hand, other potentially deleterious effects have been reported on resveratrol. In an independent study, resveratrol was able to inhibit the expression of cytochrome P450 3A4 (CYP3A4) (Deng et al., 2014), an enzyme that participates in the metabolism of many drugs whose inhibition may have significant implications when resveratrol is employed concomitantly with other drugs. For instance, resveratrol can interact with calcium channel agonists (Chai et al., 2017), immunosuppressants (Klonowska-Szymczyk et al., 2017), antihistaminic (Bedada et al., 2016), and anti-arrhythmic drugs (Stephan et al., 2017), affecting their biological activities and producing potentially severe side effects. Likewise, it has been shown that resveratrol may produce DNA damage (Liu et al., 2017) and interrupt cell cycle (Rüweler et al., 2009) that may result in a dangerous and an unwanted effect in healthy cells. Additionally, due to its chemical structure, resveratrol can act as an agonist or antagonist for estrogen receptors in several cell types (Gehm et al., 1997; Bhat et al., 2001b).

Some studies have shown that resveratrol may reduce the expression and activity of COX-1 and COX-2 enzymes, which result in decreased levels of PG-2 (Szewczuk et al., 2004; Tang et al., 2006; Zykova et al., 2008). Taken together, these findings led to the conclusion that the chronic use of resveratrol could lead to harmful gastrointestinal effects (Guha et al., 2010).

As noted, most therapeutic benefits of resveratrol are linked to its antioxidant, anti-inflammatory, neuroprotective, and cardioprotective activities (Bo et al., 2013; Carrizzo et al., 2013; Mokni et al., 2013; Zhang et al., 2018). Paradoxically, the abovementioned studies also point out a prooxidant action in cytotoxicity induced by high doses of resveratrol. Thus, it is feasible to speculate that both the dose of resveratrol and the redox state of its target site could determine either a beneficial or a toxic effect on cell viability and proliferation. This differential response could be of a particular interest in cancer where a selective cytotoxic effect in malignant cells is desirable. Consequently, despite the potential beneficial effects of resveratrol in cancer, further studies are needed to confirm their safety profile and to determine optimal therapeutic doses for every specific type of cancer.

## Resveratrol as a Nanodrug

Nanoparticles are one type of drug delivery system increasingly exploited to overcome the limitations associated with the use of standard drug preparations, such as low bioavailability, instability, high-dose requirement, poor pharmacokinetics, and rapid first pass metabolism (Mudshinge et al., 2011). Multiple studies have shown that resveratrol encapsulated in diverse nanoparticles provides the protection for resveratrol against UV light *via* improving bioavailability in different targets (Summerlin et al., 2015). Furthermore, resveratrol-loaded liposomes have shown to be effective anti-proliferative agents in brain cancer cells such as U-87 MG cells through inhibiting cell growth and inducing apoptosis in nude mice (Teskač and Kristl, 2010; Duarte et al., 2015; Summerlin et al., 2015; Jhaveri et al., 2018; Latruffe and Vervandier-Fasseur, 2018; Lian et al., 2019). In addition, the combination of resveratrol and 5-fluorouracil- (5-FU) loaded ultradeformable liposomes in an *ex vivo* study showed the ability to penetrate and deposit in the deep porcine skin layer. In this respect, both drugs work as anticancer drugs by promoting apoptosis (Cosco et al., 2015). In another study, resveratrol nanoformulation combined with dequalinium exerted an anticancer activity in two cancer cell lines (resistant A549/cDDP lung cells and non-resistant A549 lung cells), *via* triggering cell apoptosis by a mitochondria pathway (Wang et al., 2011). A group of researchers found that resveratrol-loaded liposomes have a higher anti-proliferative activity on U-87 MG cells and also inhibit the tumor growth in nude mice (Jhaveri et al., 2018). Additionally, as human serum albumin (HSA) nanoparticles are becoming a non-toxic drug delivery system, and thus their inclusion for drug nanoencapsulation was also addressed in *vitro* (human liver cancer HepG2 cells) and *in vivo* (H22 tumor-bearing mice). In this regard, in a study, the HSA system was applied to the resveratrol study. Interestingly, it was found that resveratrol-loaded HSA combined with folic acid slowed down the drug release at the injection site, whereas HSA encapsulated resveratrol without folic acid led to a considerably higher accumulation of resveratrol in tumor. In addition, resveratrol-loaded HSA nanoparticles merged with folic acid was 6-fold more bioavailable than the application of free polyphenol (Ackova et al., 2019; Chaudhary et al., 2019; Lian et al., 2019; Santos et al., 2019). In another study, it was observed that folic acid-conjugated resveratrol in combination with docetaxel nanoparticle significantly enhanced the level of apoptotic PC cells by 30.9% and 65.9%, respectively. In addition, nanoparticles of folic acid + resveratrol + docetaxel momentously decreased the levels of anti-apoptotic (BCL-XL and BCL-2) genes and COX-2, NF-kB, p65, and pro-apoptotic (BAK and BAX) while a decrease in the survival rate of cancer cells and an enhancement of the cleaved caspase-3 expressions were reported after the folic acid-conjugated docetaxel formulation (Singh et al., 2018).

In lymph node carcinoma of the prostate (LNCaP) cells, polymeric nanoparticles encapsulating resveratrol significantly lowered the cell viability (IC_50_ = 15.6 ± 1.49 and IC_90_ = 41.1 ± 2.19 μM). Multiple mechanisms participated in this case, such as the induction of apoptosis, cell cycle arrest at G1/S, externalization of phosphatidylserine, DNA damage, ROS production, and mitochondrial membrane potential loss (Nassir et al., 2018). In an *in vitro* study with human HT-29 colorectal cancer cells, omega-3 polyunsaturated fatty acids (PUFA) encapsulated solid-lipid nanoparticles (SLN) with resveratrol showed an inhibitory effect on cell growth and cell invasion (Serini et al., 2018). Casein micelles incorporated with resveratrol in MCF-7 breast cancer cells considerably decreased the tumor volume and growth biomarkers (El-Far et al., 2018). In another study, resveratrol-loaded lipid-core-nanocapsules exerted an anticancer effect in HT29 cancer cells by remarkably inducing cell apoptosis (~36%) (Feng et al., 2017). Similarly, resveratrol-loaded nanocapsules reduced cell viability, tumor growth and volume, and prevented metastasis and pulmonary hemorrhage in murine melanoma cells (Carletto et al., 2016). Lipid-polymer hybrid nanoparticles co-delivered with docetaxel and resveratrol significantly inhibited tumor proliferation (Song et al., 2018). In other studies, the cytotoxic role of encapsulated resveratrol in solid-lipid layer against the proliferation of breast cancer cell line MDA-MB-231 was also addressed, reporting a decrease in cell viability, the promotion of apoptosis, and the cell cycle arrest at G0/G1 (Medina-Aguilar et al., 2016). A study performed by other authors revealed that the encapsulation of resveratrol in an SLN dose-dependent manner suppressed cell proliferation, induced cell death, and allowed the cell cycle arrest at G0/G1 (Yip and Reed, 2008; Wang et al., 2016). In MDA-MB-231 cells, this encapsulation also enhanced the cell cycle arrest and downregulated the cyclin D1 expression in cancer cells (Jiang et al., 2017).

Taken together and despite the promising results in preclinical studies, the replication of the potential of resveratrol in clinical studies has been hampered because of its instability, short half-life, poor solubility, and low bioavailability. Indeed, when taken orally, resveratrol is rapidly metabolized to form glucuronides and sulfates and excreted, thus failing in human clinical trials (Sanna et al., 2013b; Siddiqui et al., 2015; Shindikar et al., 2016). In this sense, to overcome these physicochemical and pharmacokinetic limitations in a clinic, several attempts have been done to develop nanotechnology-based approaches to achieve adequate bioavailability and enhance efficacy at a tolerable dose (Table 1).

**Table 1:** Nanoformulations of resveratrol for cancer therapy.

**Formulation/details**	**Tested system**	**Experimental results**	**References**
Nanoparticles mPEG–PCL based nanoparticles	C6 glioma cells	• Increased cell death, cytotoxicity and intracellular ROS levels production compared to free resveratrol	Shao et al. (2009)
Resveratrol-loaded poly(ε-caprolactone) nanocapsules	Murine melanoma cell and mice model	• Inhibited cell growth and induced cell death • Reduced tumor volume and increased necrotic area and inflammatory infiltrate	Carletto et al. (2016)
Resveratrol-capped gold nanoparticles(Size: 22.28 ± 2.98 nm in diameter)	Human breast cancer cells	• Inhibited breast cancer cell progression by influencing the matrix metalloproteinase, cyclooxygenase-2, nuclear transcription factor-κB, activator protein-1, phosphoinositide 3-kinase/Akt (PI3K/Akt) and extracellular signal-regulated kinase	Park et al. (2016)
Resveratrol-doxorubicin-loaded gold nanoparticles (resveratrol_GnanoparticleS) (Average size and zeta potential: 35 nm and −21.2 mV)	Glioma carcinoma cell line	• Enhanced anticancer activity • IC_50_ value for doxorubicin loaded resveratrol-Gnanoparticles and free doxorubicin are 4 μg/mL and 6 μg/mL, respectively	Mohanty et al. (2014)
Zein/pectin core-shell nanoparticles (size ≈235 nm in diameter and contain resveratrol content of 10.2%, w/w)	Human hepatocarcinoma Bel-7402 cells	• Exhibited higher antiproliferative activity (IC50 = 17.6 μg/mL, 77.2 μM) as compared to free resveratrol (IC50 = 25.6 μg/mL, 112.0 μM)	Huang et al. (2017)
Radiolabeled resveratrol-loaded gold nanoparticles	HT29 colon cancer cells and hepatocellular carcinoma bearing animal model	• Cancer cell internalization for 99mTc-Res-Au nanoparticle was significantly higher than that of 99mTc-Au nanoparticle and 99mTc-resveratrol. • Gradual rise in target to nontarget uptake over time was observed following i.v. administration of 99mTc-Res-Au nanoparticle to colon tumor bearing rats	Kamal et al. (2018)
Liposomes Ultra-deformable liposomes (Resveratrol and 5-fluorouracil co-loaded)	SK-MEL-28 cells and Colo-38 cells	• High ability to block cell proliferation in G1/S, modifying the action of 5-fluorouracil and increasing the activity of resveratrol	Cosco et al. (2015)
Chitosan (CTS) modified liposomes, and coated by gold nanoshells (GNS@CTS@Res-lips)	HeLa cells	• Efficient on-demand pH/photothermal-sensitive drug release and improved drug cellular uptake and cytotoxicity	Wang et al. (2017)
Liposomes	PTEN-CaP8 cells and PTEN knockout mice	• Inhibited cell growth and induced apoptosis in PTEN-CaP8 cells • Downregulated p-Akt, cyclin D1, mTOR, and AR • Decreased prostatic adenocarcinoma with significant raise in curcumin concentration when co-administered with resveratrol	Narayanan et al. (2009)
Cyclodextrin cyclodextrin-based nanosponges (size between 400 to 500 nm)	HCPC-I cells	• Improved *in vitro* release and stability as compared to plain drug • Higher toxicity effects compared to free resveratrol	Ansari et al. (2011)
Nanoemulsion (lipid based nanoemulsifying resveratrol)	MCF-7 breast cancer cells	• Enhanced cytotoxicity	Pund et al. (2014)
Other approaches Lactobionic/folate dual-targeted amphiphilic maltodextrin-based micelles(resveratrol and sulfasalazine)	HepG-2 liver cancer cell	• Dual-targeted micelles enhanced cytotoxicity *via* binding to overexpressed folate and asialoglycoprotein receptors and showed improved cellular uptake • *In vivo*: Reduced liver/body weight ratio *via* stimulation of apoptotic enzyme, 3 and suppression of the VEGF (tumor angiogenic marker)	Anwar et al. (2018)

## Application of Resveratrol in Nanomedicine: Preclinical and Clinical Evidence

### Resveratrol-Loaded Nanoparticles

Several studies have shown that nanoparticle-mediated delivery can be a useful approach in improving the bioavailability of resveratrol. For example, Guo et al. (2010) in the treatment of an implanted ovarian tumor xenograft mouse with saline, free RVs, and resveratrol-loaded bovine serum albumin (BSA) nanoparticles (*i.p*.; 50, 100, and 200 mg/kg body weight) once a week for 4 weeks found that nano-resveratrol increased resveratrol concentrations in the ovary by 1.8-fold and retarded ovarian cancer growth dose-dependently when compared to free resveratrol. The inhibition rates of tumor growth by free and nanoresveratrol at 200 mg/kg body weight were 46% and 62%, respectively, without causing weight loss (Guo et al., 2010). In addition, the incorporation of resveratrol in SLN led to a reduction in cell proliferation, which has been shown to be beneficial in preventing skin cancer. The resveratrol-SLN (size < 180 nm) also showed an increased solubility, stability, and intracellular delivery (Teskač and Kristl, 2010).

In 2012, Sanna et al. have developed cationic chitosan- (CS-) and anionic alginate- (Alg-) coated poly(d,l-lactide-*co*-glycolide) nanoparticles loaded with resveratrol. The nanoparticles, spherical in shape and of size ranging from 135 to 580 nm, were efficient at delivering resveratrol through a controlled release. The findings also showed that the encapsulation of resveratrol provided significant protection against light-exposure degradation, warranting its potential for cancer therapy. The same team of researchers have also attempted to evaluate the effect of polymeric nanoparticles encapsulating resveratrol (nanoresveratrol) on human PC cells. As main findings, the authors stated that at different concentrations (10–40 μM), nanoresveratrol led to a significantly elevated cytotoxicity in PC, DU-145, and LNCaP cell lines compared to free resveratrol. The results proved that nano-resveratrol is consistently sensitive toward both the hormone-sensitive LNCaP cells and androgen-independent DU-145 PC cell lines (Sanna et al., 2013b). Another study showed that polymeric nanoparticles encapsulating resveratrol-induced apoptosis in LNCaP cells mediated by the cell cycle arrest at the G1/S phase, phosphatidylserine externalization, DNA nicking, loss of mitochondrial membrane potential, and ROS generation. Nanoresveratrol also exerted a markedly higher cytotoxicity in LNCaP cells as compared to free resveratrol at all tested concentrations (Nassir et al., 2018). In another study, Karthikeyan et al. (2013) reported that resveratrol-loaded gelatin nanoparticles enhanced the activity on NCI-H160 cells (LC cells) by reducing cell variability and mitochondrial membrane potential and elevating cytotoxicity, intracellular ROS levels, and DNA damage. HPLC analysis showed that resveratrol-gelatin nanoparticles have a greater bioavailability and half-life than free resveratrol (Karthikeyan et al., 2013). Jung et al. (2015) have investigated the *in vitro* and *in vivo* anticancer effects of newly developed resveratrol-loaded polyethylene glycol–polylactic acid (PEG–PLA; MW 5000-5000) polymer nanoparticles. As main findings, the treatment of CT26 colon cancer with 40 and 20 μM of nanoresveratrol for 72 h significantly reduced the cell number to 5.6% and the colony forming capacity to 6.3% while increasing apoptotic cell death and ^18^F-FDG uptake, along with the reduction in ROS levels. In addition, when CT26 tumor-bearing mice received intravenously nanoresveratrol at a dose of 100 mg/kg two times per week for 3 weeks, a reduction in tumor growth was stated as compared to the control group (Jung et al., 2015). Additionally, Wang et al. (2017) suggested that resveratrol-loaded SLNs (resveratrol-SLNs) were more capable at hindering the human breast cancer cell proliferation and inducing cell death in comparison with free resveratrol. The resveratrol-SLNs have shown a notable inhibitory potential in cell invasion, suggesting that resveratrol-SLN may be a promising therapeutic agent for breast cancer treatment (Wang et al., 2017).

### Resveratrol-Loaded Liposomes

Liposomes-based nanoformulations were the first to enter clinical trials, despite in recent years, several investigations have focused on the feasibility and efficacy of resveratrol-loaded liposomes in targeting cancer cells. For example, Wang et al. (2011) evaluated the effectiveness of mitochondrial targeting resveratrol modified with a dequalinium PEG-distearoylphosphatidyl ethanolamine conjugate to overcome multidrug resistance. The resveratrol-loaded liposomes were able to induce apoptosis in both non-resistant and resistant cancer cells by decreasing mitochondrial depolarization, releasing cytochrome c, and raising the caspase-9 and−3 activities. In addition, a notable anticancer effect was shown by resveratrol-loaded liposomes in xenografted resistant A549/cDDP cancers in nude mice and tumor spheroids by deep penetration into the core (Wang et al., 2011). Some years later, Meng et al. (2016) used the synthesized liposomes coloaded with resveratrol and paclitaxel for drug resistance reversal in breast cancer cells. The average size of the liposome was 50 nm, with the encapsulated efficiencies of more than 50%. As main findings, the authors stated that liposomes showed a pronounced cytotoxicity against the drug-resistant MCF-7/ADR tumor cells and an improvement in bioavailability and tumor retention of the liposome in mice with a drug-resistant tumor (Meng et al., 2016).

In an independent study, resveratrol was coloaded with 5-FU in a single PEGylated liposome to test their synergistic effects on an NT8e head and neck squamous carcinoma cell line. It was observed that the co-encapsulation with 5-FU greatly reduced the amount of resveratrol required to attain 50% cell death (5.2 vs. 31 mM) (Mohan et al., 2014). Similarly, other researchers have addressed the effects of quercetin and resveratrol co-encapsulated in liposomes against inflammatory/oxidative responses associated with skin cancer. Liposomes potentiated the anti-ROS activity in fibroblasts and improved tissue damage, with a significant reduction of oedema and leukocyte infiltration in a mouse model of skin lesion (Caddeo et al., 2016).

Transferrin (Tf) is a serum glycoprotein that takes part in the transfer of iron into growing cells by binding with a Tf receptor (TfR). Cancer cells have a relatively higher expression of TfRs to meet this elevated requirement for iron. Thus, the ability of TfR to internalize *via* clathrinid-mediated endocytosis, coupled with its high levels of expression in cancer cells make it ideal for targeting selective drug delivery to malignant cells (Daniels et al., 2006; Engelberg et al., 2018). Tf-targeted resveratrol-loaded liposomes (Tf-resveratrol-L) increased cytotoxicity and induced higher levels of apoptosis accompanied by a boost in caspases 3/7 activity in glioblastoma (GBM) cells when compared to free resveratrol or resveratrol-PEGylated liposomes. In addition, Tf-resveratrol-L also revealed to be more effective in inhibiting tumor growth and in improving the survival in an xenograft mouse model of GBM (Jhaveri et al., 2018).

### Cyclodextrins

Some researchers have also addressed the effect of cyclodextrins loaded with resveratrol. For example, Berta et al. (2010) investigated the effect of resveratrol complexed with two 2-hydroxypropyl-β-cyclodextrin formulations (cream and mouthwash) on 7,12-dimethylbenz[a]anthracene-induced hamster oral squamous cell carcinoma (OSCC) cell line (HCPC I) and in an animal model. Formulations led to an augmentation in the anti-proliferative activity in a dose and time-dependent manner. In experimental models, the formulations have prevented oral pre-neoplastic lesions and OSCC appearance and growth and showed improved efficacy (Berta et al., 2010). In another investigation, a resveratrol sulfobutyl ether-ß-cyclodextrin complex prepared in a 1:1 ratio by freeze drying to assess its influence on drug aqueous solubility and anticancer efficacy against breast cancer cell line (MCF-7 cells) and led to a strong improvement in the aqueous solubility of resveratrol, from 0.03 to 1.1 mg/ml at 25°C and improved anticancer potential while the cyclodextrin molecule without resveratrol had no effect on cell viability (Venuti et al., 2014). In another study, Lu et al. (2012), evaluated the cytotoxicity of resveratrol/β-cyclodextrin (β-CD) and resveratrol/β-CD/2-hydroxypropyl-β-cyclodextrin complexes in cancer (HeLa and Hep3B) and healthy (human umbilical vein endothelial cells [HUVEC]) cell lines. As main findings, it was observed that the complexes exerted a high cytotoxicity in both cancer cell lines, especially for Hep3B, and showed no detrimental effects on normal cells.

## Resveratrol Nanoencapsulations: Looking at Stability and Bioavailability

Plant-derived polyphenols have been increasingly reported as remarkable compounds for the treatment of neoplastic diseases. In particular, the use of resveratrol has received much attention for its potential beneficial effects in preventing the process of carcinogenesis (Delmas et al., 2006; Boocock et al., 2007; Jeandet et al., 2013). Resveratrol is a phytoalexin produced by different plant species, which, like many other low molecular weight antimicrobial compounds, is synthesized and accumulated by plants as a way to protect them from invaders (Ingham, 1976). Through its marked antioxidant effects, resveratrol counteracts cardiovascular and neoplastic pathologies. Among the cell targets of resveratrol, many transmembrane proteins were identified, e.g., human lymphocyte Kv1.3 potassium channels (Teisseyre and Michalak, 2006) and ABC transporters, such as P-glycoprotein, multidrug resistance-associated protein 1 (Wesołowska et al., 2009).

The popular theory of the ability of resveratrol to increase lifespan has been investigated in some studies conducted in yeasts (Howitz et al., 2003), worms, and fruit flies, to whom notable effects were listed (Wood et al., 2004). Nevertheless, the doses of resveratrol used in these studies may incur imponderable side effects in humans (Brown et al., 2010; Poulsen et al., 2013). Furthermore, it would be impossible to consume sufficient amounts of resveratrol with food, given the modest content of the compound present in it. Indeed, the amount of resveratrol present in wine, one of the richest foods containing this compound, depends on the grapes used in the production process, as well as on the processing and fermentation processes: the highest values are around 3 mg/L for red wines, which means that taking 50 mg of resveratrol per day, one should drink over 15 L of red wine per day (Adrian et al., 2000). Moreover, extraction techniques also affect, in a marked extent, the total amount of resveratrol extracted, and due to their low content in plants, also there is a difficulty in developing effective extraction processes and inevitable environmental problems for the possible chemical synthesis; therefore, there is a need to optimize microorganism-based processes for the large-scale production of resveratrol (Jeandet et al., 2018; Thapa et al., 2019). In addition, the application of resveratrol is strongly hampered by its limited solubility, low stability, and degradability both *in vitro* and *in vivo* (Das et al., 2008). On the other hand, resveratrol has a high solubility in PEG 400 (over 370 g/L) and alcohol (almost 88 g/L), but it is poorly soluble in soybean oil (0.14 g/L) and practically insoluble in water (only 0.05 g/L) (Robinson K. et al., 2015). Moreover, the low bioavailability of resveratrol following oral administration narrows down its application in biomedicine (Amri et al., 2012). These issues make it essential to develop alternative ways of administering resveratrol to its intake with food.

In recent years, innovative nanotechnology-based approaches have been used to solve these problems *in vivo*. Therefore, nanoformulations and the transport of bioactive compounds have started to attract the attention of pharmaceutical, functional food, and nutraceutical industries. Several novel nanometric delivery systems for bioactive compounds, ranging from nanoemulsions to SLNs, nano-dispersions, and self-assembling lipidic and biopolymeric systems, have been explored (Davatgaran-Taghipour et al., 2017; Jampilek et al., 2019; Ashrafizadeh et al., 2020). On one hand, the treatment with resveratrol nanoformulations offers many advantages in terms of greater solubility, stability, and protection from oxidation and a greater bioavailability; on the other hand, this method of administration must deal with the problems typically encountered by the circulation of nanostructures in biological fluids. Among others, the size of nanoparticles, the chemical nature of their surface, and other chemical–physical traits can constitute an obstacle to circulation and distribution in the tissues and cells of body. In addition, the high surface area per volume unit of nanoparticles allows its surface to be functionalized with specific ligands, to obtain the targeted delivery of active ingredients to specific cells. Furthermore, nanoparticles can be coated with hydrophilic polymeric materials capable of not stimulating the immune system during their transportation through the bloodstream, thus increasing their half-life *in vivo*.

Since the first demonstrations of the beneficial properties of resveratrol have been established, considerable efforts have been made to study and increase its stability *in vitro* and *in vivo* due to the possibility of dependency of a greater bioavailability of this compound. Indeed, resveratrol has a reasonable stability in acidic medium (up to pH 6), whereas its degradation starts at neutrality and continues quickly at pH around 8–9 (Robinson K. et al., 2015). In addition, the *trans* form can undergo isomerization to the *cis* form when heated or exposed to UV radiation, but such isomerization seems to be negligible *in vivo* as only trace amounts of *cis*-resveratrol can be detected when orally administered to rats and mice (Yu et al., 2002). As explained, resveratrol nanoencapsulation can improve the stability of this polyphenol, providing an effective barrier against environmental factors that can cause chemical degradation. For example, when the stability of resveratrol was measured by the conversion rate of *trans*-resveratrol to its *cis*-resveratrol isomer, the stability of resveratrol-loaded nanoparticles prepared using chitosan and γ-poly(glutamic acid) was increased by around 28% in comparison with non-encapsulated resveratrol (Chung et al., 2020). Excellent stability was also achieved with the oligonucleotide anti-miR21 and resveratrol-loaded mesoporous silica nanoparticles conjugated with hyaluronic acid for the treatment of gastric carcinoma *in vitro* (Hu et al., 2019). This high stability can be attributed to the hydrophilic hyaluronic acid coating on the surface of the mesoporous silica nanoparticle loaded with resveratrol.

One of the aspects of a paramount importance in the preparation of nanoencapsulated products concerns the total biodegradability of the matrix used to carry the active principle. From this point of view, polymers built from intermediates in a metabolic pathway offer clear advantages. Indeed, poly (lactic-co-glycolic acid) (PLGA) is a complex that is applied in many therapeutic devices approved by the USA-FDA thanks to its biodegradability and biocompatibility. Briefly, PLGA is synthesized by the copolymerization of two different cyclic monomers of lactic acid and glycolic acid; in the body, it undergoes hydrolysis to produce the original monomers which, under physiological conditions, are by-products of various metabolic pathways (Abdul Rahim et al., 2019). The most feasible reasons for the increased efficacy of these resveratrol nanoparticles are the selective endocytosis of PLGA nanoparticles and the enhanced resveratrol release. Thus, the functionalization of nanoencapsulated PLGA constitutes a promising strategy for the protection and effective delivery of anticancer agents. Resveratrol-loaded PLGA nanoparticles can increase their efficacy when conjugated with PEG (probably the best solvent for resveratrol). With these nanoformulations, the release of resveratrol did not appear to be affected by physiological changes in the pH of the medium *in vitro*. In addition, the release of resveratrol was slower compared to free resveratrol (Sanna et al., 2013b).

On the other hand, an enhanced systemic circulation time and a short biological half-life of resveratrol were achieved using poly(D,L-lactide-co-glycolide)–D-α-tocopheryl PEG 1000 succinate blend nanoparticles (resveratrol-PLGA-B nanoparticles). Pharmacokinetic studies have shown a prolonged systemic circulation of resveratrol-PLGA-B-nanoparticles up to 36 h, with ~18 times higher plasma half-life than the free resveratrol solution (Vijayakumar et al., 2016a).

An interesting method of the stabilization and delivery of resveratrol nanoparticles is plasma protein binding. As referred previously, HSA can interact with a large variety of drugs, mainly by binding to one or two major sites in HSA (Peters, 1995). This makes this protein an important regulator of the pharmacokinetic behavior of many drugs. The resveratrol-protein binding is mainly by H-bonding with polypeptide C=O, C-N, and NH groups (N' soukpoe-Kossi et al., 2006). Thus, HSA can be considered as a good carrier for the transport of resveratrol *in vivo*. In this regard, a study was conducted, in which paclitaxel (a powerful chemotherapy) was co-loaded with resveratrol and coated with BSA. Although HSA and BSA are the two proteins with different characteristics, they have some analogies in binding certain molecules (Gelamo et al., 2002). Taken together, the ability of both for the binding of resveratrol makes them valuable tools to assist in protecting resveratrol from degradation, increasing bio-availability, and improving intracellular penetration and control delivery (Rezende et al., 2019; Tabibiazar et al., 2019).

## Toxicity of Nanoencapsulated Resveratrol

### *In vitro* Studies

*In vitro* studies have been widely used for the initial screening of the potential toxicity of nanoparticle formulations encapsulating resveratrol (Table 2). Cytotoxicity assays have been widely reported, in which the safety of “blank,” that is, the nanoparticle formulation without resveratrol, is investigated. It has been found that the formulations generally cause an insignificant cytotoxicity to the relevant cancer cell lines, in addition to those using D-alpha-tocopheryl polyethylene glycol succinate (TPGS). For example, piperine-loaded mixed micelles made of Poloxamer 407 and TPGS (Zhao et al., 2019), TPGS-coated nanoparticles (Vijayakumar et al., 2016b), and PLGA-TPGS blend nanoparticles (Vijayakumar et al., 2016a) have been reported to elicit cytotoxicity, with the latter two in a dose-dependent manner. On the other hand, P127/TPGS mixed micelles proved to be safe, up to a relatively high dose (Hao et al., 2017). This is due to the fact that TPGS is cytotoxic *in vitro* and *in vivo* (Youk et al., 2005; Constantinou et al., 2012; Neophytou et al., 2014; Neophytou and Constantinou, 2015).

**Table 2:** Nanoencapsulated resveratrol formulations and their relevant *in vitro* and *in vivo* safety studies.

**Nanoparticle description**	**Study purpose**	**Type of cancer**	***In vitro* safety**	***In vivo* safety**	**References**
Peptide lipid CDO and sucrose laurate L126 liposomes encapsulating resveratrol;PS ~140 nm with narrow distribution range;spherical;EE >90%;ZP ~40 mV	Improve resveratrol physicochemical properties and delivery to effectively treat breast cancer	Breast cancer	MCR-5 and MCR-7 cells MTT cytotoxicity assay; up to 64 μm and 48 h • blank: did not affect survival of both cell lines • selective cytotoxicity against MCR-7 cells unlike free resveratrol Apoptosis assay (up to 64 μm and 48 h) • blank: low level though increased slightly with concentration Dialysis method up to 84 h • rapid then slow and sustained release	Male BALB/c nude mice (4–6 wk) IV dose up to 25 mg/kg six times in 2-day interval • no significant change in dehydration, locomotor impairment, anorexia, behavioral abnormalities or other toxicity-related changes • significantly lower effect on weight loss and histological abnormalities in heart, liver, spleen, lung and kidney than resveratrol alone • did not alter BUN, AST and ALT	Zhao et al. (2019)
P127/TPGS mixed micelles encapsulating resveratrol, functionalized with FA; PS ~20 nm; PDI 0.098; spherical; EE ~99.67%	Improve water solubility and targeted tumor site accumulation and decrease rapid metabolism	Breast cancer	MCF-7 cells; CCK-8 cytotoxic assay (up to 48 h and 300 μg/mL) • blank: no significant until relatively high concentration Dialysis method; up to 100 h • nanoparticles showed a slower and sustained-release profile	Sprague-Dawley rats (220–250 g); IV dose equivalent to 23.1 mg/kg resveratrol; up to 8 h • pharmacokinetics: higher MRT and AUC due to slow release and clearance avoidance Kunming mice (18–22 g); IV dose of 10 mg/kg resveratrol; up to 2 h; • tissue distribution: decreased accumulation in the heart and kidneys	Hao et al. (2017)
Piperine-loaded mixed micelles made of Poloxamer 407 and TPGS encapsulating resveratrol;PS 195.00 ± 1.00 nm; smooth and spherical; EE 76.50 ± 2.01%; ZP 17.99 ± 0.65 mV	Improve solubility, oral bioavailability and anticancer activity	Breast cancer	MCF-7 cells; SRB cytotoxic assay; up to 25 μg/mL and 48 h; • blank: GI_50_: 21.67 ± 0.51 mg/mL; slight toxicity due to TPGS	Male Wistar albino rats (200-240 g); oral dose equivalent to 30 mg/g resveratrol; up to 8 h; • pharmacokinetics: higher C_max_ so slower release. Female Wistar albino rats (200-240 g); oral dose equivalent to 30 mg/g resveratrol; up to 15 d; • histology: no significant brain, heart, lung, liver, kidney, stomach tissue toxicity	Jadhav et al. (2016)
Halloysite nanotubes loaded with resveratrol and coated with/out PEI-PSS/PAH or PRM/DXS; EE ~99.7%	Evaluate properties of the proposed strategy	Breast cancer	MCF-7 cells; MTT cytotoxicity assay; up to 25 mM and 96 h; -blanks: all did not decrease cell viability. Release kinetics in phosphate buffer; up to 48 h; • halloysite nanotubes loaded with resveratrol showed slow and sustained release.	N/A*	Vergaro et al. (2012)
Mesoporous silica KIT-6, MCM-41, KIT-6-NH2 and MCM-41-NH2 encapsulating resveratrol; more resveratrol dispersed on MCM-41 than KIT-6 carrier; EE 7-76%	Evaluate the properties of proposed nanoparticle	Breast cancer	MCF-7 cells; MTT cell cytotoxicity assay; up to 1 mg/ml and 24 h; • blanks: all carriers non-toxic. Release kinetics in simulated body fluid; up to ~18 h; • slower release	N/A	Latifi and Sohrabnezhad (2018)
Chitosan-gellan nanofibers encapsulating resveratrol;d 166 ± 37 and 291 ± 41 nm depending on the chitosan-gellan ratio;fiber morphology;EE: 86 ± 6%	Evaluate the characteristics of the proposed strategy and potential for gastrointestinal region delivery	Colorectal cancer	HT29 cells; MTT cytotoxicity assay; up to 30 μg/mL 72 h; • -blank (Chitosan: gellan at 90:5% w/v): no significant.	N/A	Rostami et al. (2019)
Sericin nanoparticles loaded with resveratrol;PS ~200 to 400 nm; PDI 0.2-0.4; monodisperse, spherical, smooth; EE ~70-75%; ZP ~-20 mV	Study the feasibility of the production of the proposed nanoparticle	Colorectal cancer	CRL-2522 and Caco-2 cells; MTT cytotoxicity assay; up to 1.0% and 24 h; • blank: no significant • selective cytotoxicity against Caco-2 cells. • Confocal imaging; up to 24 h; • no acute toxic damage; evident cell division. • Dialysis method; up to 72 h; • rapid then slow and sustained release	N/A	Suktham et al. (2018)
Glyceryl behenate-based solid lipid nanoparticles encapsulating resveratrol;PS 248.30 ± 3.80 nm; spherical and smooth; PDI 0.277 ± 0.017; EE 33.93%; LC 3.08%; ZP −25.49 ± 0.49 mV	Investigate the brain targeting ability of the proposed strategy	Glioma	C6 cells; MTT cytotoxicity assay; up to 250 μg/mL and 72 h; • blank: not significant. Dialysis method; up to 24 h; • rapid then sustained release.	Female adult Wistar rats (150–200 g); IP dose of 5 mg/kg; 90 min; • higher brain accumulation.	Jose et al. (2014)
mPEG–PCL based nanoparticles encapsulating resveratrol; PS 78.3 ± 7.9 nm; PDI 0.14 ± 0.04; smooth and spherical; EE 91 ± 4.6%; LC 19.4 ± 2.4%; ZP 6.5 ± 1.4 mV	Evaluate the glioma anticancer function of the proposed strategy	Glioma	C6 cells; MTT cytotoxicity assay; up to 32 μM equivalent to resveratrol and 48 h; • blank: not significant. Dialysis method; up to ~120 h; • rapid then sustained release	N/A	Shao et al. (2009)
PLGA lipid nanoparticles conjugated with ICG and FA encapsulating resveratrol; PS 92.8 ± 2.1 nm; d 104.5-121.1 nm; well-defined sphere; EE (resveratrol) 65.6 ± 4.7%; EE (ICG) 38.4 ± 4.8%; ZP −8.4 mV−47.3 mV	Evaluate for highly sensitive dual-imaging guided tumor targeted therapy	Glioma	U87 cell; CCK-8 cytotoxicity assay; up to 100 μg/mL and 24 h; • blank: not significant. Apoptosis assay; up to 30 μg/mL and 24 h; • blank: not significant. Dialysis method; up to 70 h; • rapid then sustained release.	U87 tumor-bearing BALB/c mice (6–8 wk); IV 200 μL at a dose of 15 mg/kg; up to 1 month; • high tumor selective accumulation, • no significant weight loss, • histology: no significant damages in the heart, liver, spleen, lungs and kidneys. -normal blood parameters including HGB, HCT, WBC, RBC, HGB, MPV, HCT, MCH, MCHC and MCV.	Xin et al. (2016)
TPGS-coated nanoparticles encapsulating resveratrol;PS 203.1 ± 14.91 nm; PDI 0.263 ± 0.12; spherical; EE 70.18 ± 5.19% ZP −10.5 ± 2.94mV	Improve systemic circulation time, biological half-life and brain passive targeting of resveratrol	Glioma	C6 cells; MTT cytotoxicity assay; up to 72 h; • blank: concentration-dependent cytotoxicity due to TPGS. Human blood; Hemolysis; up to dose equivalent to 100 μg/mL resveratrol and 8 h; • within limits. Platelet aggregation; up to dose equivalent to 100 μg/mL resveratrol and 2 h; • not significant. Dialysis method; up to 48 h; • rapid then sustained release.	Charles Foster's rats (150–200 g); IV dose of 2 mg/kg equivalent to resveratrol; up to 48 h; • higher brain accumulation.	Vijayakumar et al. (2016b)
PLGA:TPGS blend nanoparticles encapsulating resveratrol; PS 175.5 ± 8.47 nm; PDI 0.147 ± 0.09; spherical; EE 61.81 ± 3.57%; ZP −16.86 ± 1.59 mV	Improve systemic circulation time and biological half-life of resveratrol	Glioma	C6 cells; MTT cytotoxicity assay; up to 72 h; • blank: concentration-dependent cytotoxicity due to TPGS. Human blood; hemolysis; up to dose equivalent to 100 μg/mL resveratrol and 8 h; • within limits. Erythrocyte membrane integrity; up to dose equivalent to 100 μg/mL resveratrol and 8 h; • not significant. Platelet aggregation; up to dose equivalent to 100 μg/mL resveratrol and 2 h; • not significant. Dialysis method; up to 48 h; • rapid then sustained release.	Charles Foster's rats (150–200 g); IV dose of 2 mg/kg equivalent to resveratrol; up to 48 h; • higher brain accumulation.	Vijayakumar et al. (2016a)
Tf-modified PEG-PLA nanoparticles conjugated with resveratrol; PS 153.3 ± 28.2 nm; d 150 nm; 32 Tf on surface; EE 78.2 ± 4.8%; LC 4.35 ± 0.15% ZP −9.6 ± 0.4 mV	Evaluate the glioma anticancer function of the proposed strategy	Glioma	C6, U87 cells and rat astrocyte cells; MTT cytotoxicity assay; up to 80 μmol/L and 72 h; -blank: not significant, -normal rat astrocytes showed higher resistance. Dialysis method; up to 48 h; -rapid then sustained release.	C6 glioma bearing male Sprague–Dawley (SD) rats (180-220 g); IP dose equivalent to 15 mg/kg resveratrol; • low accumulation in brain normal tissue, • deposition in heart, liver, spleen and kidneys; high liver accumulation but did not impair function.	Guo et al. (2013)
HSA nanoparticles encapsulating resveratrol, conjugated with folate; PS 102.1 ± 4.9 nm; PDI 0.001; approximately spherical and connected to each other; EE 98.36%; LC 14.66%	Evaluate the potential for targeted cancer therapy in hepatocellular carcinoma	Liver cancer	• HepG2 cells; MTT cell viability assay; up to 48 h; • blank: N/A. • Dialysis technique; up to 25 h; • nanoparticles showed a slower release profile.	Sprague–Dawley rats (weight, 180 ± 20 g); IV dose equivalent to 6 mg/kg resveratrol per day for 2 wk; • slight weight increase; no toxic effect on organs, • immune organ index: no significant change.	Lian et al. (2019)
Gelatin nanoparticles encapsulating resveratrol by glutaraldehyde;d 294 nm; PDI 0.295;spherical; EE 93.6%; ZP −18.6 mV	Evaluate the anticancer activity	Lung cancer	NCl-H460 cells; MTT cytotoxicity assay; up to 50 μg and 24 h; • blank: N/A. • Release kinetics in PBS buffer; up to 54 h; • rapid then controlled release. • Fresh blood; erythrocyte aggregation assay; 1:1 drug: erythrocyte ratio; 1 h incubation; • no effect.	N/A	Karthikeyan et al. (2013)
HSA encapsulating resveratrol, functionalized with RGD; PS 120 ± 2.6 nm; homodisperse and spherical; EE 62.5 ± 4.21%	Evaluate the potential for targeted cancer therapy in pancreatic tumor	Pancreatic cancer	PANC-1 cells; CCK-8 cytotoxicity assay; up to 24 h and 200 μg/mL; • blank: not significant. Human red blood cells; hemolysis assay; up to 200 μg/mL and 3 h; • not significant. High colloidal stability and UV stability.	Balb/c nude mice (4–5 wk); IV dose equivalent to 5 and 10 mg/kg resveratrol; 35-day treatment; • no significant weight decrease, • histology: no significant heart, liver, spleen, lung and kidney toxicity.	Geng et al. (2017)
β-CD nanosponges encapsulating resveratrol and oxy-resveratrol; PS 213.4 ± 2.45 nm and 220.3 ± 7.24 nm; PDI 0.32 ± 0.02 and 0.29 ± 0.08; uniform spherical; EE 77.73% and 80.33%; ZP 23.6 ± 0.25 mV and 22.3 ± 0.90 mV	Improve aqueous solubility and stability	Prostate cancer	• DU-145 cells; MTT cell viability assay; up to 96 h and 100 μg/mL; • dose-dependent toxicity • blank: no significant. • Dialysis method; up to 100 h; • oxy-resveratrol nanoparticles showed more uniform and slow-release profile compared to its native form. • UV lamp method; up to 120 m; • resveratrol and oxy-resveratrol nanoparticles showed 2-fold and 3-fold higher photostability than their native forms.	N/A	Dhakar et al. (2019)

* *N/A – not applicable;*

^**^AST, aspartate amino transferase; ALT, alanine aminotransferase; AUC, area under curve; BUN, blood urea nitrogen; CCK-8, cell counting kit-8; CD, cyclodextrin; CD, 1,2- bis-myristyloxyamidopropyl ornithine; d, diameter; DXS, dextran sulfate sodium salt; EE, encapsulation efficiency; FA, folic acid; HCT, hematocrit; HGB, hemoglobin; HAS, human serum albumin; ICG, indocyanine green; IP, intraperitoneal; IV, intravenous; LC, loading capacity, MCH, mean corpuscular hemoglobin; MCHC, mean corpuscular hemoglobin concentration; MCV, mean corpuscular volume; mPEG, methoxypolyethyleneglycol; MPV, mean platelet volume; MRT, mean residence time; MTT, 3-(4,5-dimethylthiazol-2-yl)-2,5-diphenyl tetrazolium bromide; nanoparticle, nanoparticle; PAH, poly(allylamine hydrochloride); PCL, polycaprolactone; PDI, PDI; PEG, polyethyleneglycol; PL, polylactic acid; PLGA, poly(d,l-lactide-co-glycolide); PRM, protamine salt; P127, Pluronic 127; PS, particle size; PSS, poly(sodium 4-styrene-sulfonate); RBC, red blood cell count; resveratrol, resveratrol; RGD, arginine-glycine-aspartate; SRB, sulforhodamine B, Tf: transferrin; TPGS, D-α-tocopheryl polyethylene glycol 1000 succinate; US, ultrasound; WBC, white blood cell count; ZP, zeta potential.

Resveratrol has been shown to induce apoptosis in different cancer cell lines and also induce the cell cycle arrest at G1/S in breast cancer cell lines. Excess TPGS can also cause toxicity due to its surfactant properties that can induce cell damage or death by increasing cell membrane permeability (Hao et al., 2017). Nevertheless, some researchers have suggested that the additional cytotoxicity imparted by the presence of TPGS can be advantageous (Vijayakumar et al., 2016a,b) as the toxicity proved to be selective for cancer cells (Neophytou et al., 2014). These findings can be endorsed by *in vivo* studies presented here, which show negligible animal toxicity. Of note, the safety of the “blank” HSA nanoparticles conjugated with folate has not been reported (Lian et al., 2019). However, it is important to highlight that cytotoxic assays in these studies were primarily intended at investigating anticancer activities in relevant cancer cell lines in nanoparticle formulations incorporated with resveratrol. Nonetheless, some studies have successfully demonstrated the toxicity of selective cancer cells in their proposed nanoparticle formulations, studying relevant normal and cancer cells: peptide lipid CDO and sucrose laurate L126 liposomes in MCR-5 and MCR-7 cells (breast cancer) (Zhao et al., 2019); sericin nanoparticles in CRL-2522 and Caco-2 cells (colorectal cancer) (Suktham et al., 2018); and Tf-modified PEG-PLA nanoparticles in C6, U87, and rat astrocyte cells (glioma) (Jhaveri et al., 2018). Importantly, it was found that the cytotoxic selectivity of the peptide lipid CDO and sucrose laurate L126 has not been shown by free resveratrol, endorsing the superior safety of the nanoparticle formulation (Zhao et al., 2019). This approach to studying cytotoxicity should be encouraged for a better *in vitro* understanding of the targeted anticancer activities of nanoparticle formulations. In addition, a few studies also investigated the toxicity of their “blanks” using apoptosis assays and were generally considered safe at low levels (Xin et al., 2016; Jhaveri et al., 2018), though peptide lipid COD and sucrose laurate 126 liposomes (Jhaveri et al., 2018) demonstrated a slight increase in apoptotic activity with concentration. Confocal imaging was also used to investigate the safety of “blank” sericin nanoparticles, where the evidence of cell division and no acute toxic damage was found in CRL-2522 and Caco-2 cells (Suktham et al., 2018). Interestingly, the photostability of β-CD nanosponges encapsulating resveratrol and oxy-resveratrol was studied using the UV lamp method, and it was found that the nanoparticle forms showed 2-fold higher photostability than their native forms (Dhakar et al., 2019). The formulation of arginine-glycine-aspartate- (RGD-) functionalized HSA nanoparticles also showed high colloidal and UV stability (Geng et al., 2017). Indeed, the stability of the nanoparticle formulation can constitute a safety issue (Onoue et al., 2014), although, unfortunately, it has not been widely explored in the retrieved literature.

On the other hand, as most formulations of a nanoparticle must be injected, the hemocompatibility must also be a concerning safety factor. For example, the hemocompatibility of TPGS-coated (Vijayakumar et al., 2016b) and PLGA:TPGS blend (Vijayakumar et al., 2016a) nanoparticle formulations was investigated through hemolysis and platelet aggregation, and both were found within the safety limits. Moreover, a hemolysis assay was studied in HSA functionalized with RGD (Geng et al., 2017), whereas erythrocyte aggregation was studied in gelatin nanoparticles encapsulating resveratrol by glutaraldehyde (Karthikeyan et al., 2013); both formulations did not cause significant safety issues. However, in general, hemocompatibility is not as widely addressed as would be expected at the experimental (*in vitro*) level, although it is crucial, as most therapeutic nanoparticle formulations that encapsulate resveratrol are designed for the injection route. Notably, piperine-loaded mixed micelles composed of Poloxamer 407 and TPGS encapsulating resveratrol are the only formulations in the retrieved literature that should be administered orally (Jadhav et al., 2016). Also noteworthy, the *in vitro* release kinetics of the different nanoparticle formulations has been widely studied, mainly through dialysis techniques, which can be used to infer partly the safety of the formulations. This is due to the facts that the rapid release of free resveratrol has been shown to cause toxicities (Ansari et al., 2011). However, with the support of *in vitro* release kinetics, various formulations have demonstrated a biphasic *in vitro* release profile, i.e., a rapid and a slow and sustained release of resveratrol, or slower release, indirectly proving their better safety profiles (Shao et al., 2009; Vergaro et al., 2012; Karthikeyan et al., 2013; Jose et al., 2014; Vijayakumar et al., 2016a,b; Xin et al., 2016; Hao et al., 2017; Jhaveri et al., 2018; Latifi and Sohrabnezhad, 2018; Li et al., 2018; Suktham et al., 2018; Dhakar et al., 2019; Lian et al., 2019; Zhao et al., 2019).

### *In vivo* Studies

The *in vivo* data of the proposed nanoparticle strategies generally support their safety (Table 2). Still, notably, although the information about the treatment dose is generally available, the frequency of which is omitted in several papers. This aspect can markedly impair the judgment of the readers about the safety of the proposed nanoparticle formulation. Most studies used healthy and/or tumor-bearing mice and/or rats in their investigations, which mostly entail weight monitoring (Xin et al., 2016; Geng et al., 2017; Li et al., 2018; Lian et al., 2019; Zhao et al., 2019) and histopathological analysis of relevant organs (Jadhav et al., 2016; Xin et al., 2016; Geng et al., 2017; Li et al., 2018; Zhao et al., 2019), where the results supported the safety of these nanoparticle formulations. In addition, on comparing nanoparticle and free resveratrol formulations in weight loss and histopathological studies, it was found that the peptide lipid CDO and sucrose laurate L126 liposomes formulation (Zhao et al., 2019) were safer. However, this useful comparison has not been considered in other studies. The researchers also took it a step further and explored a range of behavioral abnormalities and other potential toxicity-related changes, e.g., dehydration, anorexia, and locomotor impairment, and no significant findings were reported (Zhao et al., 2019). Appropriate blood testing has also been employed by several studies to address the potential nephrotoxicity (Zhao et al., 2019), hepatotoxicity (Li et al., 2018; Zhao et al., 2019), and hemotoxicity (Xin et al., 2016; Li et al., 2018) of their formulations. In this way, the immune organ index was used by one study, exploring the potential of HSA nanoparticles encapsulating folate-conjugated resveratrol in hepatocellular carcinoma, where no significant changes were found (Lian et al., 2019).

Additionally, *in vivo* pharmacokinetics and biodistribution studies can also provide useful insights into the safety of nanoparticle formulations. As discussed above, the slow release of resveratrol is advantageous to avoid adverse effects as already demonstrated in the formulation of mixed micelles P127/TPGS by MRT and AUC (Hao et al., 2017) and in the piperidine-loaded mixed micelles made of Poloxamer 407 and TPGS by C_max_ (Jadhav et al., 2016). On the other hand, biodistribution studies have shown that the accumulation of formulations in organs can indirectly inform the possibilities of off-target side effects. For instance, P127/TPGS mixed micelles functionalized with folic acid have been shown to decrease the accumulation in the heart and kidneys than free resveratrol (Hao et al., 2017), thereby potentially decreasing the associated side effects of resveratrol. Furthermore, higher targeted and selective organ/tumor accumulation was also demonstrated in glyceryl behenate-based SLN (Jose et al., 2014), PLGA lipid nanoparticles conjugated with indocyanine green (ICG) and folic acid (Xin et al., 2016), TPGS-coated nanoparticles (Vijayakumar et al., 2016b), PLGA:TPGS blend nanoparticles (Vijayakumar et al., 2016a), and lipid microbubbles conjugated with ICG and folic acid (Li et al., 2018).

## Conclusion

Resveratrol displays an anticancer activity at various stages of carcinogenesis. It has distinct mechanisms, contributing to the induction of autophagic cell death and apoptosis, and the inhibition of angiogenesis, metastasis and modification in cancer cell metabolism, thus affecting cancer in the momentous stages of carcinogenesis. Additionally, resveratrol exhibits chemoprotective effects in cancer therapy, lowering the risk of chemotherapy-associated side effects. However, the potential negative effects of resveratrol concerning the biphasic dose-response, cytotoxicity, and interaction with some drugs are also an issue.

Oral administration of resveratrol has proved to be inadequate. Therefore, an application of nano-delivery systems has been investigated in several studies. The review of the available literature indicates that nanotechnology-based approaches, including resveratrol-loaded nanoparticles and liposomes and resveratrol, incorporated in cyclodextrin nanoformulations have proved to be sufficient methods of administering resveratrol. In this respect, it has been demonstrated that resveratrol nanoformulations protect resveratrol from degradation caused by environmental factors, as well as enhancing the stability, solubility, bioavailability, and control delivery of resveratrol, ensuring its efficiency. Importantly, nano-delivery systems loaded with resveratrol exhibit a slow release of resveratrol at the injection site, which is considered a great advantage associated with a lower risk of adverse effects. Moreover, the selective cytotoxicity of resveratrol nanoformulations is limited to cancer cells, contributing nanotechnology-based approach the advantage over free resveratrol. More studies investigating safety, especially selective cytotoxicity, biocompatibility, dose, frequency of application, and stability of nanoformulations of interest, along with for different types of cancer are necessary.

## Author Contributions

CQ, ZM, EK, AT, AKi, GS, MFM, DL, AKo, JW, HS, GL-G, MDP-A, HC, AR, PZ, OS, MI, HE, US, and RK: conceptualization. CR, NC-M, AFAR, MK, AS, and JS-R: validation investigation—data curation writing—all authors; review and editing. All the authors read and approved the final manuscript.

## Conflict of Interest

The authors declare that the research was conducted in the absence of any commercial or financial relationships that could be construed as a potential conflict of interest.

## Publisher's Note

All claims expressed in this article are solely those of the authors and do not necessarily represent those of their affiliated organizations, or those of the publisher, the editors and the reviewers. Any product that may be evaluated in this article, or claim that may be made by its manufacturer, is not guaranteed or endorsed by the publisher.
